# CT‐Guided Cryoablation Achieves Significantly Higher Local Tumor Control in Sub‐Solid Lung Tumors Compared With Solid Tumors: A Retrospective Cohort Study

**DOI:** 10.1111/1759-7714.70253

**Published:** 2026-02-20

**Authors:** Tongyin Zhang, Qiaoyu Xu, Haoyu Li, Yuwan Hu, Haoran Du, Zhenguo Huang, Yanyan Xu, Meng Yang, Hongliang Sun

**Affiliations:** ^1^ Department of Radiology China‐Japan Friendship Hospital Beijing China; ^2^ Department of Radiology, Beijing Chao‐Yang Hospital Capital Medical University Beijing China; ^3^ Peking University China‐Japan Friendship School of Clinical Medicine Beijing China; ^4^ Graduate School, Chinese Academy of Medical Science and Peking Union Medical College Beijing China; ^5^ National Center for Respiratory Medicine; State Key Laboratory of Respiratory Health and Multimorbidity; National Clinical Research Center for Respiratory Diseases; Institute of Respiratory Medicine, Chinese Academy of Medical Sciences; Department of Pulmonary and Critical Care Medicine, Center of Respiratory Medicine China‐Japan Friendship Hospital Beijing China

**Keywords:** cryoablation, CT, hemoptysis, lung tumor, pneumothorax

## Abstract

**Objective:**

Cryoablation has proved to be an effective method to cure lung cancer based upon existing research, thus has been widely used in clinical practice. This pilot study aimed to evaluate the efficacy and safety of CT‐guided cryoablation for the treatment of different lung tumors, including solid and sub‐solid lung tumors.

**Methods:**

This retrospective study enrolled a total of 77 patients with lung tumors, comprising 46 solid tumors and 31 sub‐solid tumors. All patients underwent CT‐guided cryoablation. The various aspects of the treatment, including procedural details, technical success rate, complications, and local tumor control were systematically evaluated.

**Results:**

The technical success rate of CT‐guided cryoablation was 100%. The procedure was well‐tolerated, with a low rate of major complications (4%). The most common complication is pneumothorax, which presents in 34.8% of patients with solid tumors as well as 51.6% of patients with sub‐solid tumors. The incidence rates of all the complications were not statistically different. The median follow‐up period was 24 months, during which local tumor control rates were 74% for solid lung tumors and 100% for sub‐solid tumors. The overall survival rates at 24 months were 100%.

**Conclusion:**

CT‐guided cryoablation appears to be an effective and safe treatment modality for different lung tumors, including solid and sub‐solid tumors. The high technical success rate, low rate of major complications, and favorable local tumor control suggest that cryoablation could serve as a valuable alternative treatment especially for the tumors manifesting as sub‐solid tumors.

## Graphical abstract

CT‐guided cryoablation is an effective and safe treatment for both solid and sub‐solid lung tumors, achieving 100% technical success. It demonstrates favorable local control, particularly for sub‐solid nodules, with a low rate of major complications.

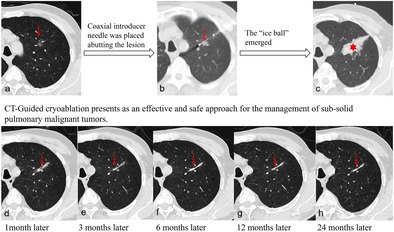

**Abbreviations**
DM: distant metastases
HLOS: hospital length of stay
LT‐PFS: local tumor progression‐free survival
MDT: multiple disciplinary team
MWA: microwave ablation
PET/CT: Positron Emission Tomography‐Computed Tomography
PGGN: pure ground‐glass nodule
PSN: part‐solid nodule
RFA: radiofrequency ablation
RLN: regional lymph node
SCLC: small cell lung cancer

## 1 Introduction

Lung cancer remains one of the most prevalent and lethal malignancies worldwide [1]. While surgery, radiation, and systemic therapies are established treatments, these modalities are often unsuitable for patients with comorbidities, multifocal disease, or limited pulmonary reserve [2, 3, 4]. This has driven development of minimally invasive ablative therapies for the management of lung tumors, with a particular focus on local ablation techniques.

CT‐guided cryoablation is one of the methods of local ablation, which has emerged as a promising technique for the local destruction of lung tumors [5]. Cryoablation can not only cause tumor necrosis and apoptosis, but also promote the release of tumor‐derived autoantigens into the blood circulation, and stimulate the host immune system to produce a good anti‐tumor immune effect against primary and metastatic tumors [6, 7, 8]. It offers several advantages, including its minimally invasive nature, precise targeting capabilities under CT guidance, and potential preservation of lung function compared with surgical resection [9, 10]. In addition to cryoablation, there are other thermal ablation techniques available, such as radiofrequency ablation (RFA) and microwave ablation (MWA). These alternative modalities also rely on thermal effects to destroy tumor cells and have been extensively validated as safe and effective treatment options in clinical practice, especially in solid tumors [11, 12]. However, these heat‐based techniques face significant limitations in sub‐solid tumors (pure ground‐glass and part‐solid lesions): the insulating effect of aerated lung parenchyma reduces thermal conduction, while adjacent vessels create heat‐sink effects—both compromising ablation completeness. Cryoablation offers distinct biophysical advantages: it preserves the collagenous architecture of the treated tissue and elicits a more pronounced post‐ablative immune response compared with the other thermal techniques [13, 14]. Furthermore, cryoablation generates visible ice balls under CT guidance (enabling margin confirmation in GGOs), and eliminates heat‐sink vulnerability by occluding microvasculature during freezing. This mechanistic advantage—coupled with reduced procedural pain and collagen preservation—positions cryoablation as a critical solution for sub‐solid tumors where other energy modalities underperform [15, 16].

Solid and sub‐solid are two distinctive forms in CT, which manifest different types in the growth of the tumor [1], evaluated on thin‐section CT (1‐mm slice thickness). Assessment window: Lung window (window width: 1500 HU, window level: −600 HU) was used for classification, as it best depicts ground‐glass and solid components. The solid means the tumor where the solid component obscures the underlying pulmonary vasculature or bronchi in all planes. The sub‐solid means the tumor with partial or complete ground‐glass attenuation that does not fully obscure the underlying vasculature/bronchi, including: pure ground‐glass nodule (PGGN), which has no visible solid component and part‐solid nodule (PSN), which has a solid component ratio (solid diameter/total nodule diameter) < 100%. This knowledge gap is clinically significant: Sub‐solid tumors (particularly adenocarcinomas in situ/minimally invasive adenocarcinomas) are increasingly detected in screening programs and often occur in patients with compromised lung function who are suboptimal surgical candidates. We therefore pose this study's core research question: Does CT‐guided cryoablation achieve differential local tumor control between solid and sub‐solid lung tumors, potentially overcoming limitations of thermal ablation in the latter group? As the first comparative analysis of cryoablation outcomes by nodule density, this retrospective cohort study evaluates technical feasibility, complication profiles and local tumor control rates of CT‐guided cryoablation across solid and sub‐solid tumors. We hypothesize that cryoablation's mechanism‐less dependent on conductive heating and better visualized may confer superior efficacy in sub‐solid tumors.

## 2 Materials and Methods

### 2.1 Study Design and Patient Selection

**FIGURE 1:**
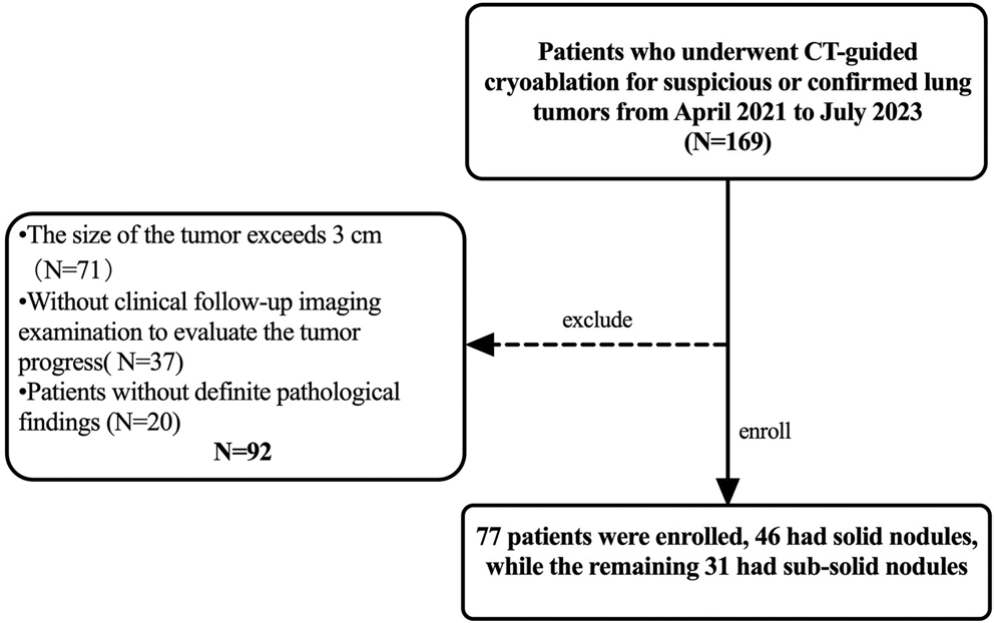
Workflow of the study.

This retrospective cohort study was approved by the Institutional Review Board of our hospital, which waived the requirement for written informed consent from the patients. The study was conducted in full compliance with the ethical guidelines and regulations of the institution. The study population consisted of 77 patients who underwent CT‐guided cryoablation for lung tumors at our hospital between April 2021 and July 2023. Eligible patients presented with either solid or sub‐solid pulmonary tumors measuring < 3 cm in diameter. According to the National Comprehensive Cancer Network (NCCN) Guidelines, cryoablation is recommended as an alternative treatment option for lung tumors measuring less than 3 cm in diameter, as this approach has been associated with improved outcomes [5]. Limiting the study population to this tumor size criteria helped to ensure consistency across the different patient groups evaluated. The inclusion criteria were as follows: (1) ≤ 3 cm highly suspicious or already confirmed malignancy pulmonary nodules based on biopsy pathology (The nodule existed persistently and multiple disciplinary team (MDT) consisted of the departments of thoracic surgery, respiratory, pathology and radiology to confirm the lesion highly suspected malignancy). (2) Clinical follow‐up with imaging examination to evaluate the tumor progress. (3) Patients who were poor candidates for surgery due to age and/or complicated with serious underlying diseases, or who refused surgical intervention. Patients with multiple intrapulmonary lesions were eligible for inclusion in our study if the target ablated lesion was ≤ 3 cm. In addition to the inclusion criteria, the following exclusion criteria were also applied: (1) Presence of severe coagulation disorders. (2) Patients with an extremely poor overall clinical condition that would preclude their ability to tolerate the procedure. (3) Lesions that were encasing the heart or major blood vessels, or did not have a safe puncture access path. (4) Patients without definite pathological findings. The workflow is listed in Figure 1. Tumors' classification was performed independently by two radiologists (blinded to pathological results, each with 10 and 15 years of relevant experience).

### 2.2 CT‐Guided Cryoablation Procedure

Prior to the cryoablation procedure, all patients underwent a pre‐procedural CT scan to thoroughly evaluate the tumor characteristics and plan the appropriate treatment approach. This assessment included measuring the tumor size, determining the precise location, evaluating the proximity to critical anatomical structures, and identifying the number of cryoablation probes required. If the lesion was in close proximity to major blood vessels, an enhanced CT scan was performed to further delineate the anatomical relationships and minimize the procedural risks.

Prior to the cryoablation procedure, patients were instructed to discontinue any anticoagulant or antiplatelet medications. During the procedure, patients were positioned in a prone, supine, or lateral orientation, depending on the location of the target lesion. The cryoablation was performed by two experienced interventional radiologists, each with 10 and 15 years of relevant experience. The procedure was conducted with the patient under moderate sedation and local anesthesia in an inpatient setting. All cryoablation procedures were performed using the commercially available Cryocare Surgical System (Endocare, Irvine, California) using argon and helium gas as the cryogen. Cryoprobes were placed under a 16‐detector‐row CT scanner (Aquilion 16; Canon Medical Systems). The specific imaging parameters were as follows: helical acquisition mode, automatic tube current modulation, 120 kVp tube voltage, and 5 mm slice thickness. To minimize radiation exposure, only the lesion area was scanned. Based on the pre‐procedural imaging assessment, the operators selected the appropriate approach to access the target lesion. The cryoprobes were then inserted into the target area, with the distance between the probe and the edge of the nodule maintained at < 0.5 cm. In cases where a single probe could not adequately ablate the entire lesion, a double‐probe technique was utilized. In cases where the target lesion could not be adequately ablated using a single cryoprobe, a double‐probe technique was employed. This “clamping freezing” approach involved the insertion of two cryoprobes on either side of the tumor. The simultaneous application of cryogenic energy from both probes created a more comprehensive ablation zone that completely encompassed the entire lesion [13]. This technique ensured that the entire target area was effectively covered, leading to improved treatment outcomes.

**FIGURE 2:**
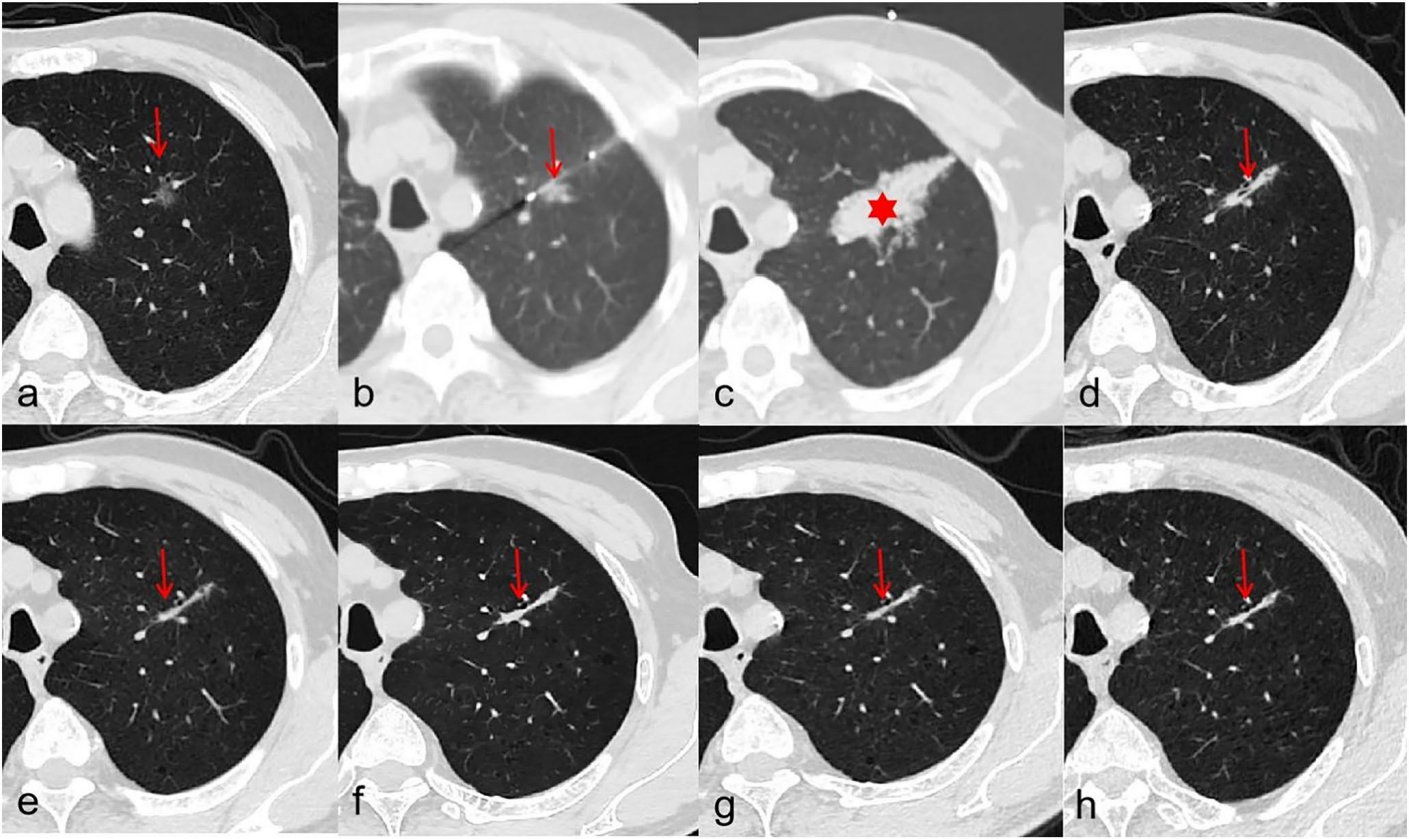
A 73‐year‐old male with a sub‐solid nodule presented a percutaneous cryoablation in the left upper lobe. (a) Before the operation, he accepted a HRCT examination to locate the lesion (arrow). (b) CT scan showing a coaxial introducer needle (arrow) which was placed abutting the lesion. (c) In the CT scan immediately after cryoablation, the ablated nodule presented an “ice ball”—a circular ground‐glass opacity (star). (d–h) The follow‐up CT scans performed 1, 3, 6, 12 and 24 months, respectively, after cryoablation. The lesion didn't recur, which showed a significantly effective result.

**FIGURE 3:**
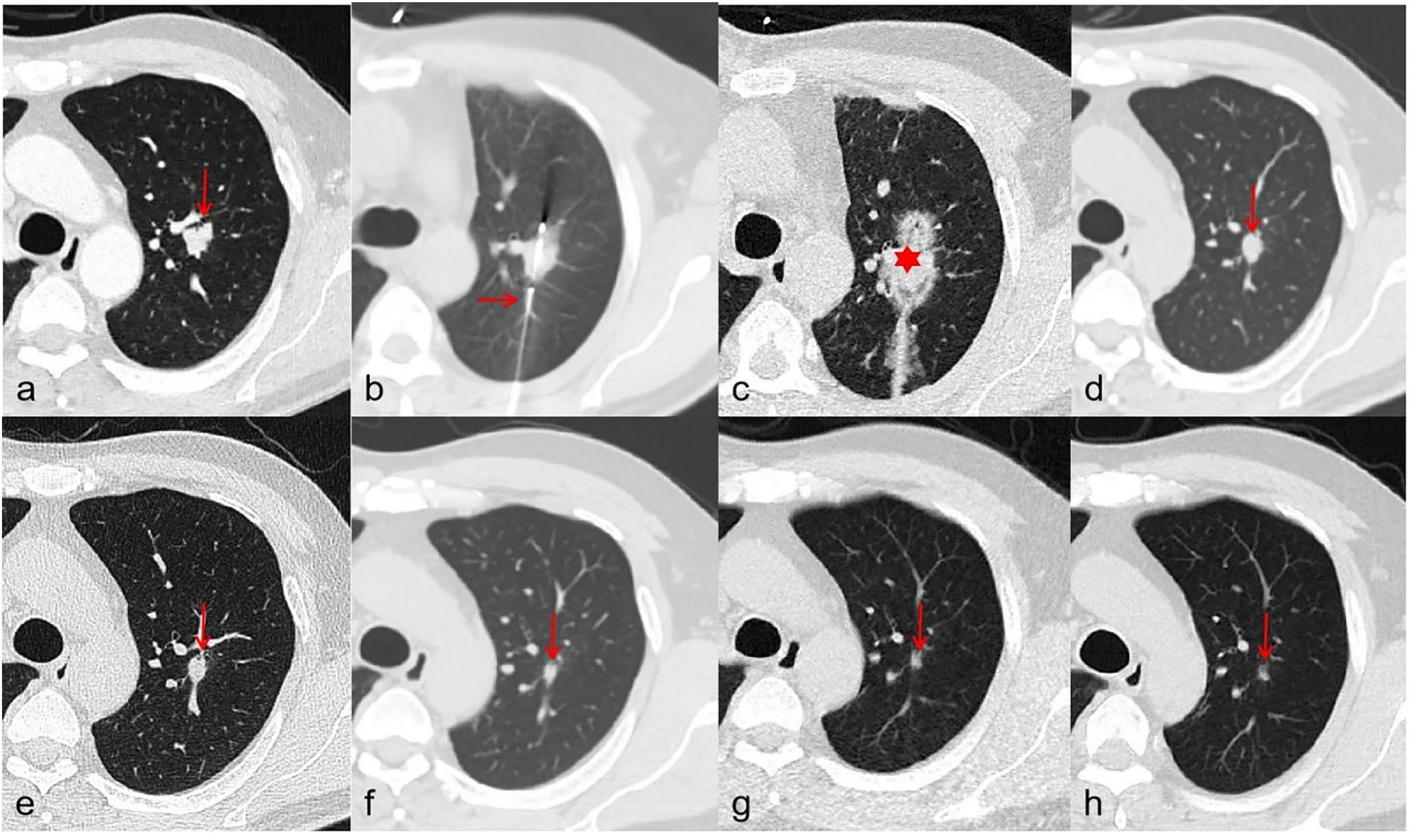
A 43‐year‐old male with a solid nodule which was confirmed to be renal carcinoma with lung metastasis presented a percutaneous cryoablation in the left upper lobe. (a) Before the operation, he accepted a HRCT examination to locate the lesion (arrow). (b) CT scan showing a coaxial introducer needle (arrow) which was placed abutting the lesion. (c) In the CT scan immediately after cryoablation, the ablated nodule presented an “ice ball”—a circular ground‐glass opacity (star). (d–h) The follow‐up CT scans performed 1, 3, 6, 12 and 24 months, respectively, after cryoablation, without tumor recurrence.

Cryoablation was performed by rapidly cooling the tissue using argon gas, which created an ice ball around the tumor. Two or three freezing–thawing cycles were applied. The argon‐helium dual‐phase cycling system was activated (argon freezing phase: needle tip temperature reaching −140°C to −160°C for 10–15 min; helium thawing phase: warming to 20°C–40°C for 3–5 min), completing 2–3 freeze–thaw cycles. Real‐time CT imaging was acquired to assess the ice ball created each time after the freezing process every 5 min. The edge of the ice ball was 1.0 cm larger than the edge of the tumor to ensure that the nodule was completely ablated to achieve a satisfactory clinical outcome (Figures 2 and 3).

Of the total patient cohort, 13 individuals underwent a synchronous biopsy procedure as part of the cryoablation treatment. For the remaining patients, the pathological diagnosis had already been established through prior biopsy.

### 2.3 Post‐Procedural Care and Follow‐Up

After the cryoablation procedure, patients were closely monitored in a dedicated recovery area for a specified period. Post‐procedural CT scans were then performed to assess the immediate technical success of the cryoablation and to detect any potential immediate complications. Treatment‐related complications were evaluated in line with the criteria set by the Society of Interventional Radiology [17, 18]. The principal complications encompassed pneumothorax, hemoptysis, and pleural effusion. Major complications pose a threat to life and necessitate extended hospitalization. In contrast, minor complications are self‐limiting and merely require a brief hospital stay for observation or treatment. Pneumothorax was graded as severe when lung compression exceeded 50%, moderate when lung compression was between 20% and 50%, and mild when lung compression was 20% or less. Hemoptysis was classified as severe if it was more than 100 mL, moderate for 10–100 mL, and mild when it was 10 mL or less. Pleural effusion was categorized as severe when it was over 1000 mL, moderate for 500–1000 mL, and mild when it was 500 mL or less.

Patients were subsequently followed up at regular intervals, with clinical evaluations and imaging studies (non‐contrast CT or PET/CT) conducted at 1–3 months after the treatment, and then every 3 months thereafter. These follow‐up assessments were aimed at closely monitoring the changes in the treated lesion and evaluating the long‐term outcomes of the cryoablation procedure. The primary endpoint was local tumor progression, which is defined as enlargement of the ablated zone accompanied by irregular enhancement or eccentric growth (≥ 20% increase in the maximum diameter of the ablated lesion (including the surrounding reactive zone) compared with the baseline scan (1 month post‐ablation) or new enhancement of the ablated lesion on contrast‐enhanced CT). While local control alone does not fully capture overall oncological outcomes. During follow‐up, regional lymph node (RLN) status was evaluated via chest CT (assessing mediastinal/hilar lymph node enlargement or enhancement), and distant metastases (DM) were assessed via whole‐body contrast‐enhanced CT (or PET‐CT for high‐risk patients) at 12 and 24 months.

### 2.4 Data Collection and Statistical Analysis

Data on patient demographics, tumor characteristics, procedural details, complications, treatment response, and overall survival were collected from electronic medical records. This included information such as the patients' age, gender, and medical history, as well as details about the tumors themselves like size, location, and growth patterns. The procedural information gathered encompassed the specific treatments administered, including any surgical interventions, radiation therapy, or chemotherapy. Complications that arose during or after the procedures were also documented. Treatment response was assessed by tracking changes in tumor size and other markers over time, and overall survival rates were calculated.

Continuous data were expressed as means with the SD or as medians with the range or interquartile range (IQR). Percentages were used to represent categorical data. To further elucidate the differences between solid and sub‐solid tumor types, Pearson's Chi‐squared test or Fisher’s exact test were utilized. A *p* < 0.05 was considered statistically significant, indicating a low probability that the differences were random. The insights gained from this comprehensive evaluation can inform future clinical decision‐making and guide the development of more targeted and effective treatment strategies.

To assess the efficacy of the cryoablation procedures performed, the researchers calculated the technical success rate. Complete ablation, where the entire tumor is destroyed, is the primary goal of the cryoablation treatment. The primary endpoint of the study was the local recurrence rate. This measures the incidence of the tumor reappearing or continuing to grow within the same location after the cryoablation treatment. To analyze the local tumor progression, the researchers utilized the Kaplan–Meier method. This statistical technique allows for the estimation of survival probabilities over time, accounting for censored data (patients who are lost to follow‐up or whose outcomes have not yet occurred). The median imaging follow‐up duration, which represents the typical length of time patients were monitored with imaging scans, was also calculated. All of these analysis were performed using the SPSS software package, version 25.0. By calculating the technical success rate, local recurrence rate, and the local tumor progression, the researchers were able to thoroughly evaluate the efficacy of the cryoablation procedures. The Kaplan–Meier analysis, in particular, provided insights into the long‐term tumor control achieved with this treatment approach. Taken together, these metrics offer a robust assessment of the clinical outcomes for patients undergoing cryoablation.

## 3 Results

### 3.1 Basic Clinical Characteristics

The basic characteristics of the eligible studies and patients are summarized in Table 1, which covers the data collected from April 2021 to July 2023. The study cohort consisted of 77 patients with solid or sub‐solid lung tumors that were highly suspicious for malignancy or had already been confirmed as malignant, all of whom underwent cryoablation treatment. Among these patients, 46 (59.7%) had solid tumors, while the remaining 31 (40.3%) had sub‐solid tumors. In the sub‐solid tumors' group, 17 patients had lesions presenting as PGGN, while another 14 had PSN. Among these 14 PSN cases, 7 patients showed lesions with a solid component accounting for more than 50% of the total tumor diameter.

**TABLE 1:** Characteristics of patients with lung tumor who underwent cryoablation.

Characteristic	Total	Patient with solid tumor	Patient with sub‐solid tumor	*p*
Patient number	77	46	31	
Sex (males)	41/77	27/46	14/31	0.243
Sex (females)	36/77	19/46	17/31	
Age (years) median (IQR)*	66.0 (56.0–71.0)	66.0 (57.0–71.0)	63.0 (55.0–71.0)	0.971
Cancer history	48/77	33/46	15/31	0.038
Previous treatment	62/77	41/46	21/31	0.020
Underlying pulmonary disease	15/77	9/46	6/31	0.982
Emphysema	7	5	2	
Interstitial lung disease	12	8	4	
Family history	0/77	0/46	0/31	

*Note:* Continuous data are presented as median (IQR) (*); Categorical data were expressed with frequencies/total patient numbers of its group.

Abbreviation: IQR, interquartile range.

### 3.2 Procedure and Complications

Table 2 and Table 3 summarize the characteristics of the cryoablation procedures and the associated complications. The tumor location, patient positioning, number of cryoablation needles used, and the duration of the freeze cycles did not show any statistically significant differences between the groups. However, the maximum axial tumor diameter was significantly larger in the solid tumor group compared with the sub‐solid tumor group (*p* = 0.002), which may also be correlated with tumor progression observed during the follow‐up period. All cryoablation procedures were successfully completed, resulting in a 100% technical success rate. The main complications observed included pneumothorax, hemoptysis, and pleural effusion. Pneumothorax was the most common complication, occurring in 34.8% of patients with solid tumors and 51.6% of patients with sub‐solid tumors. Notably, 15.2% and 19.4% of patients experienced hemoptysis in the solid tumors' group and sub‐solid tumors' group, respectively. While the incidence rates of pleural effusion were 13.0% and 22.3% in the solid tumor's group and sub‐solid tumor's group. Two patients with solid tumors and one patient with sub‐solid tumors required chest tube drainage for the management of pneumothorax. No rare complications, such as air embolization, bronchopleural fistula, pulmonary artery pseudoaneurysm, or tumor needle seeding, were detected during the follow‐up period. The median hospital length of stay (HLOS) was 2 days, and no patients required admission to the intensive care unit.

**TABLE 2:** Basic date of the nodule and procedure.

Characteristic	Total	Patient with solid tumor	Patient with sub‐solid tumor	*p*
Tumor location				0.198
Right upper lobe	22/77	11/46	11/31	
Right middle lobe	4/77	2/46	2/31	
Right lower lobe	20/77	13/46	7/31	
Left upper lobe	12/77	5/46	7/31	
Left lower lobe	19/77	15/46	4/31	
Patients' position				0.060
Supine	20/77	8/46	12/31	
Prone	36/77	26/46	10/31	
Lateral	21/77	12/46	9/31	
Maximum axial tumor diameter (cm, mean ± SD)	1.54 ± 0.71	1.74 ± 0.72	1.24 ± 0.59	0.002
Number of cryoablation probes				0.749
1	61/77	37/46	24/31	
2	16/77	9/46	7/31	
Freeze duration per tumor (min) median (IQR)*	33.0 (30.0–38.0)	33.0 (30.0–38.3)	33.0 (31.0–37.0)	0.489

*Note:* Continuous data are presented as mean ± SD or median (IQR) (*). Categorical data were expressed with frequencies/total patient numbers of its group.

Abbreviations: IQR, interquartile range; SD, standard deviation.

**TABLE 3:** Complications and outcome of procedures and hospital length of stay.

	Total	Patient with solid tumor	Patient with sub‐solid tumor	*p*
Pneumothorax (%)	41.6	34.8	51.6	0.343
Mild	26/77	12/46	14/31	
Moderate	3/77	2/46	1/31	
Severe	3/77	2/46	1/31	
Pleural effusion (%)	16.9	13.0	22.3	0.500
Mild	11/77	5/46	6/31	
Moderate	2/77	1/46	1/31	
Severe	0/77	0/46	0/31	
Hemoptysis (%)	16.9	15.2	19.4	0.155
Mild	8/77	6/46	2/31	
Moderate	5/77	1/46	4/31	
Severe	0/77	0/46	0/31	
Air embolism	0	0	0	
Primary technical success	77/77	46/46	31/31	
Hospital length of stay (day) median (IQR)*	2.0 (1.0–3.0)	2.0 (2.0–3.0)	2.0 (1.0–2.5)	0.034

*Note:* Continuous data are presented as median (IQR) (*); Categorical data were expressed with frequencies/total patient numbers of its group.

Abbreviation: IQR, interquartile range.

### 3.3 Pathological Findings

By analyzing the 77 nodules of patients, all of them were diagnosed as malignant through biopsy pathological results. Thirteen patients underwent synchronous cryoablation followed by biopsy, of which 10 had sub‐solid tumors. As for the patients with solid nodules, 11 were diagnosed as adenocarcinoma, 3 were diagnosed as squamous cell carcinoma, 3 were diagnosed as SCLC, and 29 were diagnosed as metastasis (the detailed breakdown of their primary tumor histologies and corresponding case numbers presented in Table 4). In patients with sub‐solid tumors, 29 were diagnosed as adenocarcinoma, 2 were diagnosed as squamous cell carcinoma. The types of malignancy are detailed in Table 4.

**TABLE 4:** Detailed types of nodule.

Pathologic result	Total	Patient with solid tumor	Patient with sub‐solid tumor
Adenocarcinoma	40/77	11/46	29/31
Squamous cell carcinoma	5/77	3/46	2/31
SCLC	3/77	3/46	0/31
Metastasis	29/77	29/46	0/31
Colorectal cancer	22	22	0
Germinoma	2	2	0
Ovarian cancer	1	1	0
Breast cancer	1	1	0
Renal carcinoma	1	1	0
Urothelium carcinoma	1	1	0
Cervical cancer	1	1	0

*Note:* Categorical data were expressed with frequencies/total patient numbers of its group.

Abbreviation: SCLC, small cell lung cancer.

### 3.4 Efficacy

**FIGURE 4:**
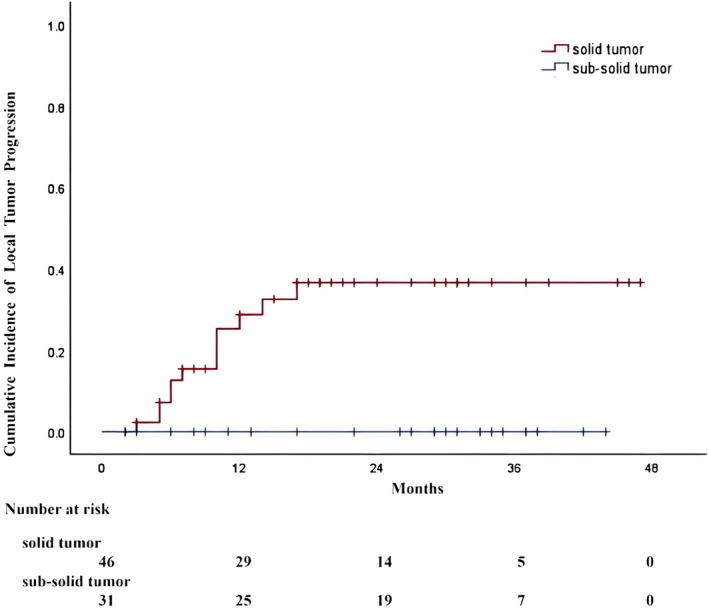
Kaplan–Meier curves depicted the cumulative incidence of local tumor progression post‐cryoablation. The ‘+’ symbols in the figure denote censored data (e.g., loss to follow‐up, event‐free at study termination). In the solid tumor group (red curve), the overall cumulative incidence was 20% at 1 year and 26% at 2 years. The sub‐solid tumor group (blue curve) demonstrated no significant progression, which was significantly lower than the solid group (*p* = 0.003).

The median imaging follow‐up duration was 24 months, ranging from 5 to 50 months. Among these, the median follow‐up time was 17 months in the solid tumor group and 29 months in the sub‐solid tumor group. All patients underwent either CT or PET/CT imaging for the evaluation of treatment response. During this follow‐up period, local progression was observed in 12 (26%) of the 46 solid tumors. In contrast, no local tumor progression or tumor seeding was identified in patients with sub‐solid tumors, which was lower than the solid tumors' group (*p* = 0.003) (Figure 4). What's more, 29 of 46 solid tumors were metastatic lesions; among these, 8 patients with pathologically confirmed rectal cancer metastases developed progression of ablated lesions during the follow‐up period. Among the other 4 patients with local progression, 3 had poorly differentiated lung squamous cell carcinoma, and the remaining 1 had lung adenocarcinoma refractory to both chemotherapy and immunotherapy. Notably, there were no procedure‐related or tumor‐related deaths reported during the follow‐up period. No regional lymph node metastasis or distant metastasis was identified prior to the occurrence of local tumor progression.

## 4 Discussion

Primary lung cancer continues to be the leading cause of cancer‐related mortality among both men and women, representing a significant threat to public health. Concurrently, the lung serves as the most prevalent site for tumor metastasis across a wide range of malignant neoplasms, especially colorectal cancer. While surgical resection plays a vital role in the treatment of primary and metastatic lung cancers, many patients are unable to undergo surgery due to advanced age, comorbidities, poor cardiopulmonary function, or personal preference. In recent years, image‐guided percutaneous lung ablation has made significant advancements and is emerging as a highly promising treatment modality, particularly the technique of CT‐guided cryoablation.

In our study, we compared the efficacy and safety of CT‐guided cryoablation for different types of lung tumors. The high technical success rate of 100% across both groups clearly demonstrates the efficacy of this image‐guided ablation technique in achieving complete tumor destruction. The most common complication observed was pneumothorax, which occurred in both the solid and sub‐solid tumor groups [19]. Notably, the risk of pneumothorax was higher in patients with underlying pulmonary conditions, such as emphysema and interstitial lung disease, despite no statistical difference in the prevalence of these conditions between the two sample groups. Additionally, repeated needle insertions during concurrent biopsy procedures also increased the likelihood of this complication [20, 21]. However, the rates of pneumothorax were not statistically different between the two tumor groups. The second most frequent complications were hemoptysis and pleural effusion, which are commonly associated with lung cryoablation [22, 23]. However, the rates of these complications were not statistically different between the groups, which was likely due to the small sample size. Patients with sub‐solid tumors may be more prone to experiencing hemoptysis, as the procedure may involve traversing more normal pulmonary parenchyma. Importantly, the overall incidence of major complications was low, at only 4%. This suggests that CT‐guided cryoablation is a relatively safe procedure, with a low risk of significant adverse events, a finding that is consistent with previous research [24, 25].

Compared with other ablation techniques like radiofrequency ablation (RFA) and microwave ablation (MWA), cryoablation is associated with less pain and better preservation of the tissue collagen architecture [26]. This makes cryoablation the preferred approach for tumors located close to the pleura [27]. The local tumor control rates observed in this study were also encouraging, with a 100% rate for malignant sub‐solid tumors and a 74% rate for solid lung tumors during the follow‐up period. While the therapeutic effect of sub‐solid tumors' group is considerably better than that of solid lung tumors' group, the disparity is statistically significant. This is likely because malignant sub‐solid pulmonary tumors tend to be relatively slow‐growing compared with malignant solid tumors [28]. As the majority of sub‐solid tumors reflect a pre‐invasive or minimally invasive histology, they typically have a better prognosis and a slower rate of progression to invasion, which is marked by the development of a solid component detectable on serial CT imaging [29, 30]. Therefore, the superior local control in subsolid nodules is primarily attributable to their indolent biological behavior rather than superior ablation performance. While a substantial proportion of tumors in the solid group consisted of highly aggressive histologies, such as small cell lung cancer (SCLC) and metastatic lesions, which differ fundamentally from primary non‐small cell lung cancer (NSCLC) in terms of biological behavior, treatment sensitivity, and therapeutic response. In our study, 8 out of 12 cases of progressive tumors were metastatic tumors, and the remaining 4 were primary tumors (3 cases of poorly differentiated lung squamous cell carcinoma and 1 case of refractory lung adenocarcinoma), which is consistent with our hypothesis. Concurrent with this pathological heterogeneity, our data confirmed a statistically significant difference in tumor size between the two groups (*p* < 0.05), with the solid tumor group having larger lesions. Notably, tumor size is a well‐established prognostic factor for lung lesions treated with cryoablation; larger tumor dimensions are associated with higher risks of incomplete ablation and subsequent local progression. This size discrepancy between the two groups may therefore represent an important confounding variable that partially contributes to the observed differences in oncological outcomes. Consequently, the local control rate for sub‐solid tumors is higher than that for solid nodules. However, these survival rates are comparable to those reported for other minimally invasive treatment modalities, such as RFA or MWA [31, 32, 33].

It is important to acknowledge the limitations of this retrospective study. This is a single‐center, retrospective study with inherent limitations in generalizability; therefore, we need multicenter, prospective studies and external validation to confirm the reproducibility of our findings. While potential selection bias may also impact the generalizability and robustness of the findings. The superior local control observed in this study may be attributable, in part, to patient selection; cryoablation was more frequently used for sub‐solid tumors, which are generally associated with indolent biology and a favorable prognosis. In addition, the small sample size reflects the relative rarity of patients meeting strict inclusion criteria, which leads to an inability to perform subgroup analysis within the solid group. In subsequent studies, we will prioritize the recruitment of a substantially larger sample size to enhance the statistical power and generalizability of our findings. Another limitation of the study was the lack of standardized use of enhanced CT imaging to evaluate blood perfusion within the tumors. Without this information, it was challenging to account for the potential impact of perfusion heterogeneity on the thermal energy deposition during the cryoablation procedures [34]. Variations in tumor vascularity and blood flow can influence the distribution of the cryogenic freezing, which may in turn affect the technical success and outcomes of the treatment [34]. Future studies should incorporate the systematic assessment of tumor perfusion, using techniques such as contrast‐enhanced CT or other advanced imaging modalities, to better understand and control for this important factor in the cryoablation process. Additionally, the follow‐up duration may not be sufficient to assess long‐term outcomes, since primary lung cancers presenting as ground‐glass nodules are known to have indolent growth patterns, and longer‐term studies are needed to fully evaluate the durability of the observed local tumor control rates. Given these limitations, further prospective studies with larger sample sizes and longer follow‐up periods are warranted to confirm these results and optimize patient selection criteria and treatment protocols for CT‐guided cryoablation of lung tumors. Such studies would help strengthen the evidence base and provide a more comprehensive understanding of the technique's efficacy and suitability for different lung tumor types and patient populations.

## 5 Conclusion

Overall, the results of this pilot study suggest that CT‐guided cryoablation is an effective and safe treatment modality for various types of lung tumors, particularly those presenting as sub‐solid tumors. Consequently, this minimally invasive technology is expected to see increased use in the comprehensive treatment of precursor lesions and early invasive subtypes in the future.

## Author Contributions

H.S. is the guarantor of this manuscript. All authors had full access to all of the study data and take responsibility for the integrity of the data and the accuracy of the data analysis and the manuscript. T.Z., H.L., Y.H., H.D., Q.X., Y.X., Z.H., M.Y., and H.S. contributed substantially to the study design, data analysis and interpretation, and writing of the manuscript.

## Funding

This work was supported by the National Key R&D Program of China, 2023YFC2508605.

## Ethics Statement

The study was conducted in accordance with the ethical guidelines and regulations of the institutional review board (IRB) of China‐Japan friendship hospital (Approval number: 2023‐KY‐013‐1).

## Conflicts of Interest

The authors declare no conflicts of interest.

## Data Availability

The data that support the findings of this study are available from the corresponding author upon reasonable request.
